# Bio-upcycling of even and uneven medium-chain-length diols and dicarboxylates to polyhydroxyalkanoates using engineered *Pseudomonas putida*

**DOI:** 10.1186/s12934-024-02310-7

**Published:** 2024-02-16

**Authors:** Yannic S. Ackermann, Jan de Witt, Mariela P. Mezzina, Christoph Schroth, Tino Polen, Pablo I. Nikel, Benedikt Wynands, Nick Wierckx

**Affiliations:** 1 Institute of Bio- and Geosciences IBG-1: Biotechnology, Forschungszentrum Jülich, Jülich, Germany; 2 The Novo Nordisk Foundation Center for Biosustainability, Technical University of Denmark, Kongens Lyngby, Denmark

**Keywords:** *Pseudomonas putida*, Metabolic engineering, Dicarboxylates, Diols, Bio-upcycling, Polyhydroxyalkanoates

## Abstract

Bio-upcycling of plastics is an emerging alternative process that focuses on extracting value from a wide range of plastic waste streams. Such streams are typically too contaminated to be effectively processed using traditional recycling technologies. Medium-chain-length (mcl) diols and dicarboxylates (DCA) are major products of chemically or enzymatically depolymerized plastics, such as polyesters or polyethers. In this study, we enabled the efficient metabolism of mcl-diols and -DCA in engineered *Pseudomonas putida* as a prerequisite for subsequent bio-upcycling. We identified the transcriptional regulator GcdR as target for enabling metabolism of uneven mcl-DCA such as pimelate, and uncovered amino acid substitutions that lead to an increased coupling between the heterologous β-oxidation of mcl-DCA and the native degradation of short-chain-length DCA. Adaptive laboratory evolution and subsequent reverse engineering unravelled two distinct pathways for mcl-diol metabolism in *P. putida*, namely *via* the hydroxy acid and subsequent native β-oxidation or *via* full oxidation to the dicarboxylic acid that is further metabolized by heterologous β-oxidation. Furthermore, we demonstrated the production of polyhydroxyalkanoates from mcl-diols and -DCA by a single strain combining all required metabolic features. Overall, this study provides a powerful platform strain for the bio-upcycling of complex plastic hydrolysates to polyhydroxyalkanoates and leads the path for future yield optimizations.

**Graphical Abstract:**

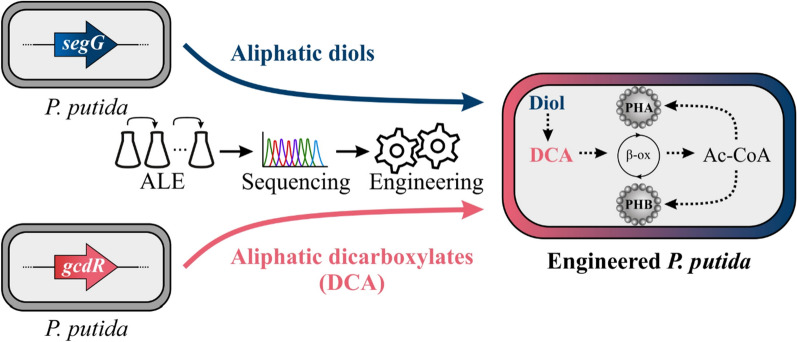

**Supplementary Information:**

The online version contains supplementary material available at 10.1186/s12934-024-02310-7.

## Background

The plastic crisis is a pressing environmental issue facilitated by an increasing plastic production that reached about 390 million metric tons in 2021, of which 90% was based on fossil raw materials. More than half of the plastics produced are polyolefins such as polypropylene, low- or high-density polyethylene, and polyesters like PET [1]. Especially mixed plastics are a major challenge for mechanical and chemical recycling as they typically require pure feedstocks or the costly purification of individual building blocks [2–4].

Bio-upcycling is a promising strategy to overcome the drawbacks of conventional end-of-life solutions [5]. This describes the process of biologically converting plastic waste into valuable products or materials through (bio-)depolymerization and subsequent microbial cultivation. Such conversion could provide better end-of-life options for hard-to-recycle polymers and composites because biology is uniquely capable to work with complex mixtures and materials [6]. Furthermore, significant efforts were invested in the past to combine enzymatic or chemical depolymerisation with microbial metabolization by using genetic and metabolic engineering [3]. For example, pyrolysis was used to produce hydrocarbon wax from PE polymers, which was subsequently oxidized to a mixture of fatty acids. This mixture could then serve as substrate for polyhydroxyalkanoate (PHA) production in *Pseudomonas* [7]. Furthermore, Sullivan et al. combined a chemical auto-oxidation step to break down the carbon bonds of high-density PE or PET with a microbial bioconversion step to further metabolize the resulted monomers into new compounds [8]. Unfortunately, both chemical and enzymatic degradation processes sometimes lead to unfavourable by-products such as toxic monomers or require harmful solvents [9]. Although in some cases it is possible to separate toxic compounds, such as aromatic diamines [10, 11], this will not always be economically feasible. Therefore, it is important to use robust microbial hosts. One promising candidate is the widely used biotechnological host *Pseudomonas putida* KT2440 [12, 13]. Besides a high tolerance to chemical stress and rapid growth, in previous studies *P. putida* was already enabled to grow on different plastic monomers such as 1,4-butanediol (BDO), adipic acid (AA), ethylene glycol, or itaconate [11, 14–17]. Moreover, *P. putida* KT2440 was engineered to serve as platform organism for the production of several value-added molecules including aromatic compounds [13], rhamnolipids [18], and medium-chain-length (mcl) polyhydroxyalkanoates (PHA), consisting of C_6_-C_12_ monomers [19–21].

Mcl-aliphatic diols, such as BDO and 1,6-hexanediol (HDO), are prevalent monomers of polyurethanes or polyesters. Previous studies enhanced metabolism of BDO in *P. putida* KT2440 [17]. A mutation in a transcriptional regulator, encoded by PP_2046, activated the downstream operon PP_2047-51, thereby greatly enhancing the rate of BDO metabolism. Since this operon encodes enzymes involved in β-oxidation, it is likely that BDO is converted to glycolyl-CoA and acetyl-CoA, although direct oxidation to succinate could not be excluded. A relevant group of intermediates within this pathway are the partly oxidized hydroxy acids (HA) such as 6-hydroxyhexanoate which is the monomer of polycaprolactone. The production of PHA from BDO by *P. putida* was successfully shown [17]. Nevertheless, several other mcl-diols including HDO can currently not be funneled into the central metabolism of *P. putida* for bio-upcycling.

Together with mcl-aliphatic diols, mcl-dicarboxylates (DCA) are mainly used for the synthesis of polyesters but also to produce polyamides and polyurethanes. Furthermore, mcl-DCA are products from chemical oxidation of longer polyolefins [8]. Growth on single mcl-DCA was already achieved with the engineered *P. putida* KT2440ge Δ*P*_*paaF*_-*paaYX*::*P*_*14g*_ Δ*psrA* (KT2440-AA) strain expressing the heterologous *dcaAKIJP* cluster from *Acinetobacter baylyi* [14]. However, metabolism of especially uneven-chain-length (ucl) DCA is still rather inefficient, especially in the case of pimelate (C_7_). An exception to this is glutarate (C_5_), which is a favorable native carbon source for *P. putida* and is metabolized through two independent pathways. One is regulated by the GntR family regulator CsiR, which induces a CoA-independent pathway with glutarate hydroxylase (CsiD) and l-2-hydroxyglutarate oxidase (LhgO) as key enzymes [22]. Furthermore, *P. putida* contains a CoA-dependent pathway, in which glutarate is activated by a CoA-transferase (PP_0159) to glutaryl-CoA and then further decarboxylated by glutaryl-CoA dehydrogenase (GcdH) to crotonyl-CoA [23]. Crotonyl-CoA can then be converted *via* acetoacetyl-CoA into two acetyl-CoA molecules.

In this study, we aimed to extend the substrate range of *P. putida* KT2440 with prevalent polyethylene and polyester hydrolysate constituents, namely mcl-diols and –DCA, using metabolic engineering and laboratory evolution. Especially metabolism of substrates of uneven chain-length is limited and needs to be addressed. The combination of unravelled pathways should result in a mutant that is able to funnel a complex polyester mock hydrolysate into its central metabolism providing it as substrate for bio-upcycling. To demonstrate such an approach, PHA and poly(3-hydroxybutyrate) (PHB) production from mcl-diols and -DCA as pure substrates and in a mock hydrolysate is envisioned. Altogether, this study leads the path for future bio-upcycling of mixed plastic hydrolysates that currently are a burden to conventional recycling.

## Methods

### Strains and culture conditions

The chemicals used in this work were obtained from Carl Roth (Karlsruhe, Germany), Sigma-Aldrich (St. Louis, MO, USA), or Merck (Darmstadt, Germany) unless stated otherwise.

All bacterial strains used in this work are listed in Table S1. Unless otherwise stated, *P. putida* KT2440 strains were cultivated for quantitative microbiology experiments in three-fold buffered (11.64 g L^−1^ K_2_HPO_4_, 4.89 g L^−1^ NaH_2_PO_4_) MSM. Pre-cultures contained 20 mM glucose. For cultivation experiments, the final concentrations of diols or dicarboxylates were C-molar equivalent to 30 mM adipate. For online growth measurements cultures were grown and analyzed with the Growth Profiler® 960 (Enzyscreen, Heemstede, The Netherlands) by image analysis. Main cultures were cultivated in transparent bottom 96-well microtiter plates (CR1496dg) with a volume of 200 µL at 30 °C and 225 rpm shaking speed.

Adaptive laboratory evolution (ALE) on HDO was performed in 96-well microtiter plates by iterative inoculation of fresh medium after the stationary phase was reached. For *P. putida* KT2440-AA, 30 mM HDO was used as sole carbon source. Since *P. putida* KT2440 did not grow with HDO as sole carbon source at the start of the ALE, 15 mM HDO and 15 mM BDO were used for the first two batches of the ALE. This concentration was shifted to 20 mM HDO and 10 mM BDO (batches 3–5), and to 30 mM HDO (batches 6–14). After ALE, single clones were isolated on LB agar plates and screened for growth on HDO as sole carbon source. The best growing strains were selected for whole genome sequencing.

Liquid cultivations with additional analysis were incubated at 30 °C, with a shaking speed of 200 rpm and an amplitude of 50 mm using Climo-Shaker ISF1-X (Kuhner Shaker, Birsfelden, Switzerland) in 500 mL non-baffled Erlenmeyer flasks with metal caps, containing 50 mL culture volume. For PHA production experiments, cells were cultivated in three-fold buffered and nitrogen-limited MSM. For this, a C:N ratio of 30:1 was used. PHB production was carried out in three-fold buffered MSM with 1 mM of cyclohexanone as inducer and 10 µg mL^−1^ gentamicin to maintain the pS6311·PHB plasmid. Cultivations were performed in 500 mL shake flasks with 50 mL of culture volume at 30 °C and 200 rpm until the stationary phase was reached.

### Plasmid cloning and strain engineering

Cloning primers were ordered as unmodified DNA oligonucleotides from Eurofins Genomics (Ebersberg, Germany) and are listed in Table S2. The Q5 High-Fidelity 2X Master Mix (New-England Biolabs, Ipswich, MA, USA) was used for the amplification of cloning fragments, while the One*Taq* Quick-Load 2X Master Mix (New-England Biolabs, Ipswich, MA, USA) was used for screening together with a pre-lysis step in alkaline PEG200 [24]. Plasmids used in this study were assembled by Gibson assembly [25] using the NEBuilder HiFi DNA assembly Master Mix (New-England Biolabs, Ipswich, MA, USA) or USER cloning [26] and are listed with more details in Table S3. In order to bestow PHB biosynthesis to *P. putida* strains, plasmid pS6311·PHB was constructed as follows. First, plasmid pS648::(sRBS)*phaCAB* was constructed in order to introduce synthetic RBSs upstream of each gene comprising the PHB operon. Such sRBSs sequences were introduced in the USER primers and *phaCAB* from *Cupriavidus necator* H16 was amplified from pS341·PHA [27]. The resulting plasmid, bearing sRBSs upstream of each of the three genes comprising the *pha* operon, was then used as template for the amplification of this construction. Lastly, pSEVA2311 [28] was used as template for PCR amplification of the ChnR/P_*chnB*_ expression system. These two USER fragments were used to assemble plasmid pS6311·PHB.

Transformation of *E. coli* with assembled DNA and purified plasmids was performed by a heat chock protocol [29]. Transformation of *P. putida* was performed by electroporation or conjugational transfer of mobilized plasmids by patch mating as described by Wynands et al. [30]. Knockouts, promoter exchanges and point mutations were obtained using either a modified pSNW2 system from Volke et al. [31] based on the pEMG system described by Martínez-García and de Lorenzo [32] or the original system with a modified protocol described by Wynands et al. [30]. Antibiotics were added to the medium as needed to support plasmid maintenance and to select for genomic recombination events (final concentration: Kanamycin sulfate 50 mg L ^−1^; Gentamicin 25 mg L^−1^). Plasmids, gene deletions and point mutations were confirmed by Sanger sequencing performed by Eurofins Genomics (Ebersberg, Germany).

### RT-qPCR

To analyze gene expression levels, RT-qPCR was performed. Therefore, pre-cultures of *P. putida* strains were used to inoculate 50 mL shake flask main cultures in three-fold buffered MSM containing either glutarate (36 mM), pimelate (25.7 mM), azelate (20 mM) or glucose (20 mM) as sole carbon source to an initial OD_600_ of 0.1. After incubation to mid-exponential growth phase, cells were harvested from 2 mL of cell culture by centrifugation (21,000 × *g* for 2 min) and immediately resuspended in 1 mL RNAlater. (Thermo Fisher Scientific, Massachusetts, USA) and stored at -20 °C until further analysis. RNA extraction was performed using the Quick-RNA Miniprep Kit (Zymo Research, Irvine, CA, USA) and cDNA was prepared from the purified RNA using the LunaScript RT superMix Kit (New England Biolabs, Ipswich, MA, USA). The expression levels of target genes were analyzed using primers designed by qPCR assay design tool from Eurofins Genomics and listed in Table S2 (*gcdH*) or in Otto et al. [33] (*rpoD*). Quantitative RT-PCR was performed using Luna Universal qPCR Master Mix (New England Biolabs, Ipswich, MA, USA) in 96-well plates by the qTOWER 2.2 (Analytik Jena, Jena, Germany). The reaction conditions were used as described in the manufacturer’s instructions. Experiments were performed in technical triplicates of biological duplicates. Gene expression levels were evaluated by comparing the Ct values of the housekeeping gene *rpoD* [34] with the Ct value of *gcdH* using the following equation:

$${\text{Gene}}\;{\text{expression}}\;{\text{level}} = {2}^{{{\text{Ct}}\left( {rpoD} \right) - {\text{Ct}}({\text{target}})}} .$$.

### Genome sequencing

Genomic DNA from selected strains was purified using a Monarch Genomic DNA Purification Kit (NEB) from an overnight LB culture. Afterwards, 1 µg of DNA was used for library preparation using the NEBNext® Ultra™ II DNA Library Prep Kit for Illumina® (New England Biolabs, Ipswich, MA, USA). The library was evaluated by qPCR using the KAPA library quantification kit (Peqlab, Erlangen, Germany). Afterwards, normalization for pooling was done and paired-end sequencing with a read length of 2 × 150 bases was performed on a MiSeq (Illumina, San Diego, CA, USA). The sequencing output (base calls) were received as demultiplexed fastq files. The data (e.g. trimming, mapping, coverage extraction) were processed using the CLC Genomic Workbench software (Qiagen Aarhus A/S, Aarhus, Denmark). For each sample, the output was mapped to the GenBank accession AE015451.2 as the *P. putida* KT2440 reference genome with further modifications for previous genetic engineering [14]. Sequencing data are deposited in the NCBI Sequence Read Archive under BioProject number PRJNA987418.

### Analytical methods

In shake flask experiments, bacterial growth was monitored as optical density at a wavelength of 600 nm (OD_600_) with an Ultrospec 10 cell Density Meter (Ge Healthcare, Little Chalfront, Buckinghamshire, United Kingdom). Online analysis of growth was measured by the Growth Profiler and analyzed using the Growth Profiler Control software V2_0_0. The corresponding green values are derived from image analysis of the image taken from the bottom of microtiter plates.

For measuring extracellular mcl-diols and DCA metabolites, samples were harvested from liquid cultivation by centrifugation (21,000 × *g* for 2 min) and the supernatant was analysed using a 1260 Infinity II HPLC equipped with a 1260 Infinity II Refractive Index Detector (Agilent, Santa Clara, California, USA). Analytes were eluted using a 150 × 7.80 mm organic acid resin column (Rezex ROA – organic acid H+ (8%), Phenomenex, Torrance, CA, USA) together with a 40 × 8 mm organic acid resin pre-column with 5 mM H_2_SO_4_ as mobile phase at a flow rate of 0.7 mL min^−1^ at 80 °C. Metabolites were quantified using HPLC-grade chemicals.

### PHA and PHB analysis  via gas chromatography

PHA and PHB quantification was performed using acidic methanolysis and gas chromatography (GC) analysis as described in Li et al. [17]. For this, cells were harvested by centrifugation at 5000 × *g* for 10 min and washed with H_2_O. Prior to analysis, samples were lyophilized overnight in a Christ LT-105 freeze drier (Martin Christ Gefriertrocknungsanlagen, Osterode am Harz, Germany). Next, 5–15 mg of lyophilized cells were mixed with 2 mL acidified methanol (15% (v/v) H_2_SO_4_) and 2 mL chloroform containing methyl benzoate as internal standard in a 15 mL Pyrex tube. The tube was sealed and incubated at 100 °C for 3 h. After cooling the tubes on ice for 2 min, 1 mL of H_2_O_MilliQ_ was added to each tube and the solution was mixed by vortexing. The phases were allowed to separate and the organic phase (lower phase) was filtered through cotton wool before further analysis.

The 3-hydroxyalkanoic acid methyl ester were quantified using an Agilent 7890 A Gas Chromatograph equipped with a HP Innowax column (30 m × 0.25 mm × 0.5 μm) and a flame ionization detector (FID). An oven ramp cycle was employed as follows: 120 °C for 5 min, increasing by 3 °C/min to 180 °C, 180 °C for 10 min. A 10:1 split was used with helium as the carrier gas and an inlet temperature of 250 °C. Commercially available 3-hydroxyalkanoic acids (C_4_-C_12_) were methylated as described above and used as standards to quantify PHA monomers.

## Results and discussion

### Engineering metabolism of aliphatic diols

**Fig. 1:**
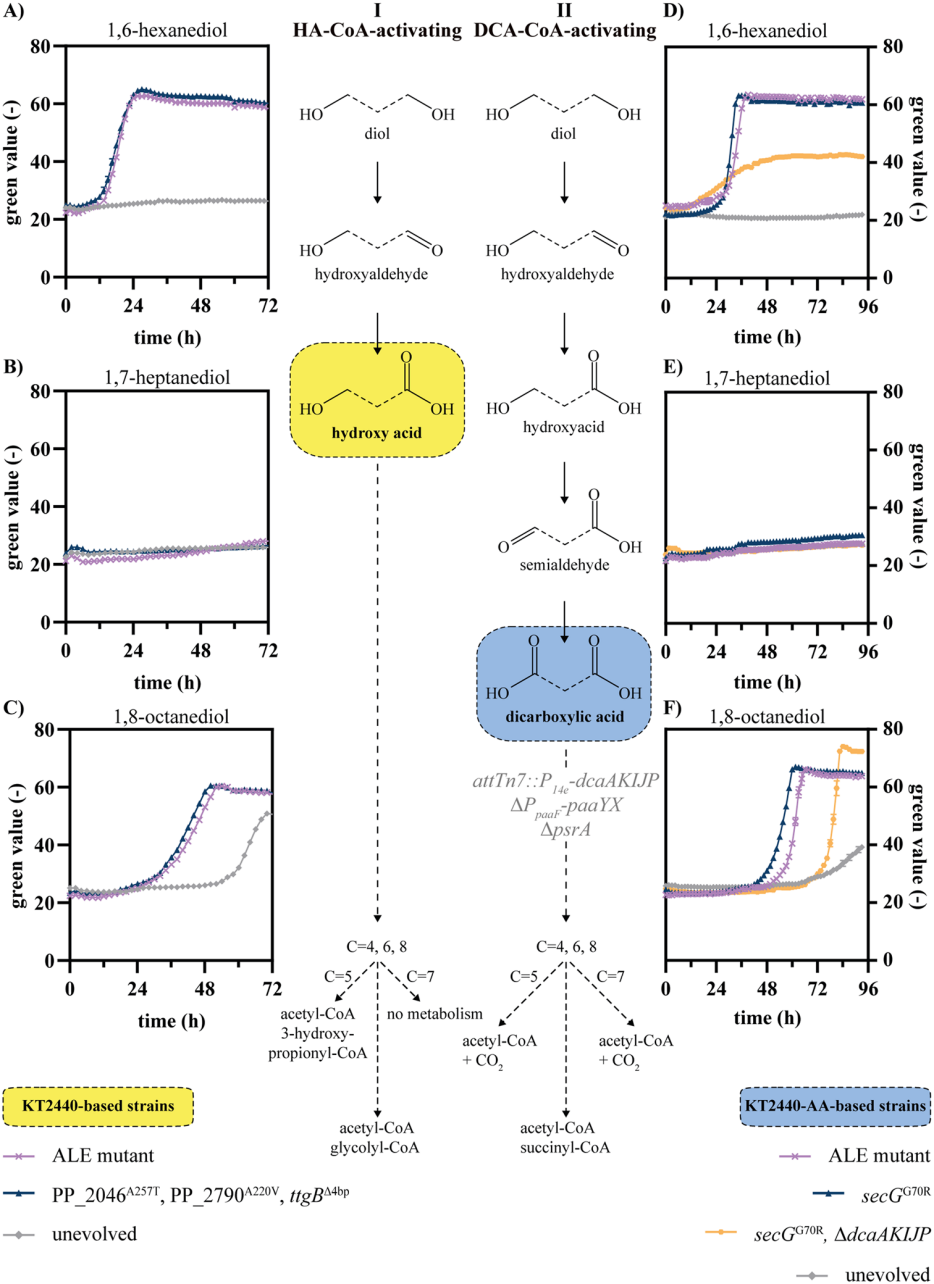
Metabolic pathways of aliphatic diols in engineered *P. putida *KT2440. *P. putida* KT2440 wild type-based strains (**A**–**C**) and *P. putida* KT2440-AA-based strains (**D**–**F**) were cultivated in mineral salts medium (MSM) supplemented with 1,6-hexanediol, 1,7-heptanediol, or 1,8-octanediol in concentrations that are C-mol equivalent to 30 mM 1,6-hexanediol. Depending on the background strain, the mcl-diols are either metabolized *via* the HA-CoA-activating (I) or DCA-CoA-activating (II) pathway which required the expression of the heterologous *dcaAKIJP* cluster in *P. putida* KT2440-AA (genomic modifications in grey). Depending on the chain-length of the diol (dashed lines), namely C = 4 (1,4-butanediol), C = 5 (1,5-pentanediol), C = 6 (1,6-hexanediol), C = 7 (1,7-heptanediol), and C = 8 (1,8-octanediol) and the respective pathway, different central metabolites are obtained. The results of single mutant cultivation are shown in Fig. S2. Growth was monitored using a Growth Profiler. Error bars indicate the standard error of the mean (*n* = 3)

Aliphatic mcl-diols are prevalent monomers in a variety of polymers such as polyesters or polyurethanes. In previous work, *P. putida* KT2440 was engineered to metabolize BDO as sole carbon source [17]. The metabolic pathway for BDO was predicted to occur via its partial oxidation to 4-hydroxybutyrate followed either by CoA-activation and subsequent β-oxidation resulting in acetyl-CoA and glycolyl-CoA, or by full oxidation to succinate. In contrast to succinate, longer chain-length DCA and thus the corresponding diols cannot be directly funneled into the central metabolism but require the heterologous β-oxidation for DCA [14]. Consequently, two different pathways might enable metabolism of aliphatic diols, in which either the partly oxidized HA (HA-CoA-activating) or the further oxidized DCA is CoA-activated (DCA-CoA-activating) (Fig. 1). As the wild type strain is not capable of metabolizing mcl-DCAs, engineering its background might only enable degradation via the HA-CoA-activating pathway. In contrast, engineering of *P. putida* KT2440-AA, which is able to metabolize mcl-DCA, could lead to degradation via both pathways.

To enable growth on HDO via the HA-CoA-activating pathway, adaptive laboratory evolution (ALE) of the *P. putida* KT2440 wild type was performed on HDO (Fig. S1). Subsequent whole-genome sequencing of ALE mutants and reverse engineering resulted in the triple mutant PP_2046^A257T^, PP_2790^A220V^, *ttgG*^∆4 bp^ that metabolized HDO and 1,8-octanediol (ODO) but not 1,7-heptanediol (Fig. 1). Interestingly, the transcriptional activator encoded by PP_2046 that was already involved in BDO metabolism, was revealed to be involved in HDO metabolism as well. Hence, HDO was likely metabolized by the HA-CoA-activating pathway encoded by PP_2047-51. Additionally, a mutation within a second regulator, more specifically a sigma factor 54-dependent sensory box protein encoded by PP_2790, was found to be involved in HDO metabolism. This regulator might activate expression of orthologs of this pathway with higher affinities for HDO than BDO. Moreover, a frameshift mutation within *ttgB* (PP_1385) encoding an efflux pump membrane protein increased growth on HDO. Possibly, the intact TtgABC efflux pump reduces intracellular HDO concentrations thereby hindering its metabolism.

In addition to the HA-CoA-activating pathway, HDO might also be metabolized by the DCA-CoA-activating pathway via adipate (AA). Therefore, *P. putida* KT2440-AA that was recently engineered to metabolize AA and other even chain-length DCA was chosen as a starting point for enabling mcl-diol metabolism. Although this strain was not able to grow on HDO as sole carbon source (Fig. 1), ALE resulted in the isolation of mutants able to metabolize 15 mM HDO within 24 h (Fig. S1). Whole-genome sequencing of the fastest-growing ALE mutant revealed two single nucleotide variants (SNV). The first SNV occurred in PP_5423 encoding a putative membrane protein causing arginine 29 to be replaced by proline (PP_5423^R29P^). The second mutation caused the exchange of glycine 70 to arginine in the protein translocase subunit SecG encoded by PP_5706 (*secG*^G70R^). Both positions are highly conserved among Pseudomonads. Reverse engineering of the unevolved *P. putida* KT2440-AA revealed that the *secG*^G70R^ mutation alone could reproduce the growth phenotype of the isolated ALE mutant. Reverse engineering of the PP_5423^R29P^ mutation enabled growth on HDO, albeit much slower and with a long lag phase. Combination of both *secG*^G70R^ and PP_5423^R29P^ in one strain did not further enhance growth compared to the *secG*^G70R^ mutant (Figure S2), indicating the mutated SecG protein as most important for HDO metabolism. SecG is an auxiliary protein that recognizes pre-protein signal sequences and builds the core of the protein translocation apparatus SecABCDEFGY [35]. Deletion of *secG* in the unevolved *P. putida* KT2440-AA mimicked the phenotype of the *secG*^G70R^ mutant on HDO as sole carbon source indicating that the SNV likely caused a loss of function (Figure S3). We speculate that this mutation could affect the subcellular localisation of oxidoreductases, thereby influencing the transport of HDO and/or its intermediates into the cytoplasm. However, global effects on other proteins, such as transporters, or signalling pathways are also conceivable but further investigations are required to unravel the exact mechanisms. Because the metabolism of 6-hydroxyhexanoate was found to not require the *secG*^G70R^ mutation, we conclude that this mutation affects the first oxidation steps of the diol to the HA (Fig. S4). In addition to DCA-CoA-activating pathway, 6-hydroxyhexanoate was also metabolized via the HA-CoA-activating pathway in the reverse engineered KT2440 wild type-based strain. Hence, 6-hydroxyhexanoate can be directed into the central metabolism via both pathways enabling future bio-upcycling processes of polycaprolactone hydrolysates.

In addition to HDO, the reverse engineered *secG*^G70R^ mutant was also able to utilize ODO as sole carbon source, whereas 1,7-heptanediol was poorly metabolized by the strain (Fig. 1). These results are in agreement with the ability of the parent strain *P. putida* KT2440-AA to metabolize the corresponding dicarboxylate suberate (C_8_) much better than pimelate (C_7_) [14]. To test whether HDO and ODO were metabolized via the DCA-CoA-activating pathway, the *dcaAKIJP* operon, enabling growth on mcl-DCA, was deleted in *P. putida* KT2440-AA *secG*^G70R^. Indeed, the *secG*^G70R^ Δ*dcaAKIJP* mutant showed decreased growth with HDO and ODO indicating that both substrates were metabolized *via* their mcl-DCA (Fig. 1). However, this indicated that the HA-CoA-activating pathway was also active in the *P. putida* KT2440-AA-based strain. Although the *secG*^G70R^ Δ*dcaAKIJP* mutant showed an increased lag-phase with ODO compared to HDO, the observed growth indicated that ODO is the favoured substrate for the HA-CoA-activating pathway. Deletion of PP_2051 encoding a 3-ketoacyl-CoA thiolase that is involved in the degradation of BDO, did not alter the phenotypes of the *secG*^G70R^ Δ*dcaAKIJP* mutant with HDO and ODO, likely due to the presence of isozymes [36] (Fig. S3). Hence, both pathways can be used to metabolize mcl-diols but they result in different central metabolites as end products namely acetyl-CoA and glycolyl-CoA for the HA-CoA-activating or acetyl-CoA and succinyl-CoA for the DCA-CoA-activating pathway (Fig. 1). In contrast to succinyl-CoA, the metabolic route for glycolyl-CoA is unknown, but a conversion to glyoxylate is likely. This can be funneled into the glyoxylate shunt [37], or it might also be metabolized via tartronate semialdehyde yielding 2-phosphoglycerate that is an intermediate of glycolysis [16]. Since degradation of glycolyl-CoA can be associated with the release of CO_2_ and consumption of NAD(P)H, the HA-CoA-activating pathway might be energetically inferior compared to the DCA-CoA-activating pathway. In addition to this, the deletion of *dcaAKIJP* within the DCA-CoA-activating pathway might enable the consolidation of a mixture containing diols and DCA to a single group of monomers. Such bioconversion using a Δ*dcaAKIJP* mutant would enhance the economic viability of monomer recycling from PE hydrolysates as not a heterogeneous mixture of monomers but only a single type of building blocks needs to be purified from the hydrolysate.

### Engineering metabolism of ucl-DCA

**Fig. 2:**
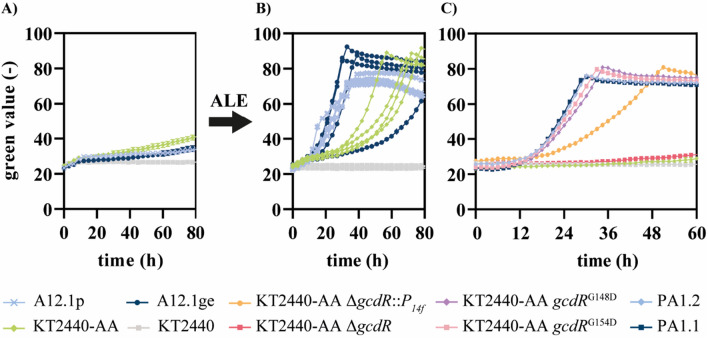
Adaptive laboratory evolution and reverse engineering for growth on pimelate. All strains were cultivated in three-fold buffered MSM containing 25.7 mM pimelate as sole carbon source. **A** Long-term cultivation of strains that are not able to grow on pimelate to induce adaptive mutations. **B** Growth of single strains which were isolated on LB agar plates after 80 h from the experiment shown in **A**. **C** Growth of reverse engineered strains based on mutations found after whole-genome sequencing of evolved *P. putida* A12.1ge strains. Growth was monitored using a Growth Profiler. Error bars indicate the standard error of the mean, but errors are sometimes so small that they are not visible behind the lines (*n* = 3)

Given that *P. putida* KT2440-AA grows very poorly on ucl-DCA, the inability of the *secG*^G70R^ mutant to metabolize ucl-diols likely stems from this downstream limitation. Hence, the next step was to optimize catabolism of ucl-DCA. Sullivan et al. demonstrated the upcycling of a DCA mixture from plastic waste containing polyesters [8]. Even when growth was enabled on the mixture containing C_4_-C_17_ DCA and all substrates were degraded over time, growth inhibition was observed on the single monomers with uneven-chain-length, especially pimelate (C_7_). This suggested a further misregulation of connecting metabolic pathways, possibly at the point of glutaryl-CoA, resulting from β-oxidation of these DCA [38]. Since it is known that pimelate cannot act as an inducer of GcdR, the absence of glutarate could explain the difference in growth between a monomer mixture and pimelate as sole carbon source [39]. To further investigate this misregulation, an evolution experiment was performed. *P. putida* KT2440-AA and the corresponding evolved strains *P. putida* A12.p and A12.1ge [14], were cultivated in MSM containing pimelate as sole carbon source to provoke stable mutations. After 70–80 h of cultivation, weak growth was detectable (Fig. 2). The cultures of all replicates were spread on LB agar plates and single colonies were re-inoculated in MSM containing pimelate as sole carbon source. This re-inoculation resulted in a significantly shorter lag phase and better growth, suggesting that stable mutations had occurred.

Whole-genome sequencing of two of the *P. putida* A12.1ge strains re-inoculated on pimelate revealed mutations in the regulator *gcdR*. One strain (PA1.2) contained a C﻿→T mutation in *gcdR* resulting in a G148D substitution, while the other strain (PA1.1) contained a C→T mutation resulting in a G154D substitution. Among Pseudomonads, both positions are highly conserved and the emerged amino acid exchanges are located in the substrate binding domain of GcdR. This LysR family regulator governs the expression of *gcdH*, encoding a glutaryl-CoA dehydrogenase, and PP_0159, encoding a family III CoA-transferase [23, 40]. Glutarate is the effector of GcdR [23, 39], but since pimelate is degraded *via* glutaryl-CoA and not glutarate, the reason for the poor growth is likely the lack of induction of *gcdH*. We hypothesized that the mutations found in *gcdR* ameliorate this lack of induction, hence enhancing growth on longer- ucl-DCA.

To investigate the impact of the regulator GcdR on the degradation of ucl-DCA, a *gcdR* knockout strain was compared to a strain harbouring the synthetic promoter *P*_*14f*_ for constitutive expression of *gcdH-*PP_0159 (Fig. 2). Since *P. putida* KT2440-AA Δ*gcdR* was not able to grow on pimelate as sole carbon source, it is likely that GcdR activates the transcription of *gcdH-*PP_0159. Growth on glutarate was not decreased by the deletion of *gcdR* probably due to the second CoA-independent degradation pathway of *P. putida* (Fig. S5). In contrast, constitutive expression of *gcdH-*PP_0159 enabled growth on pimelate, but at a lower rate than in the two evolved strains, indicating that activation of the native promoter mediated by the mutated GcdR is stronger than the expression obtained using a constitutive synthetic promoter. This was confirmed by genomic insertion of the mutations encoding the amino acid exchanges found in the evolved strains. Growth of these reverse engineered strains was much better compared to the constitutive *P*_*14f*_ expression, almost completely mimicking the growth phenotype of the evolved strains (Fig. 2).

### SNVs in *gcdR* may cause changes in ligand binding

**Fig. 3:**
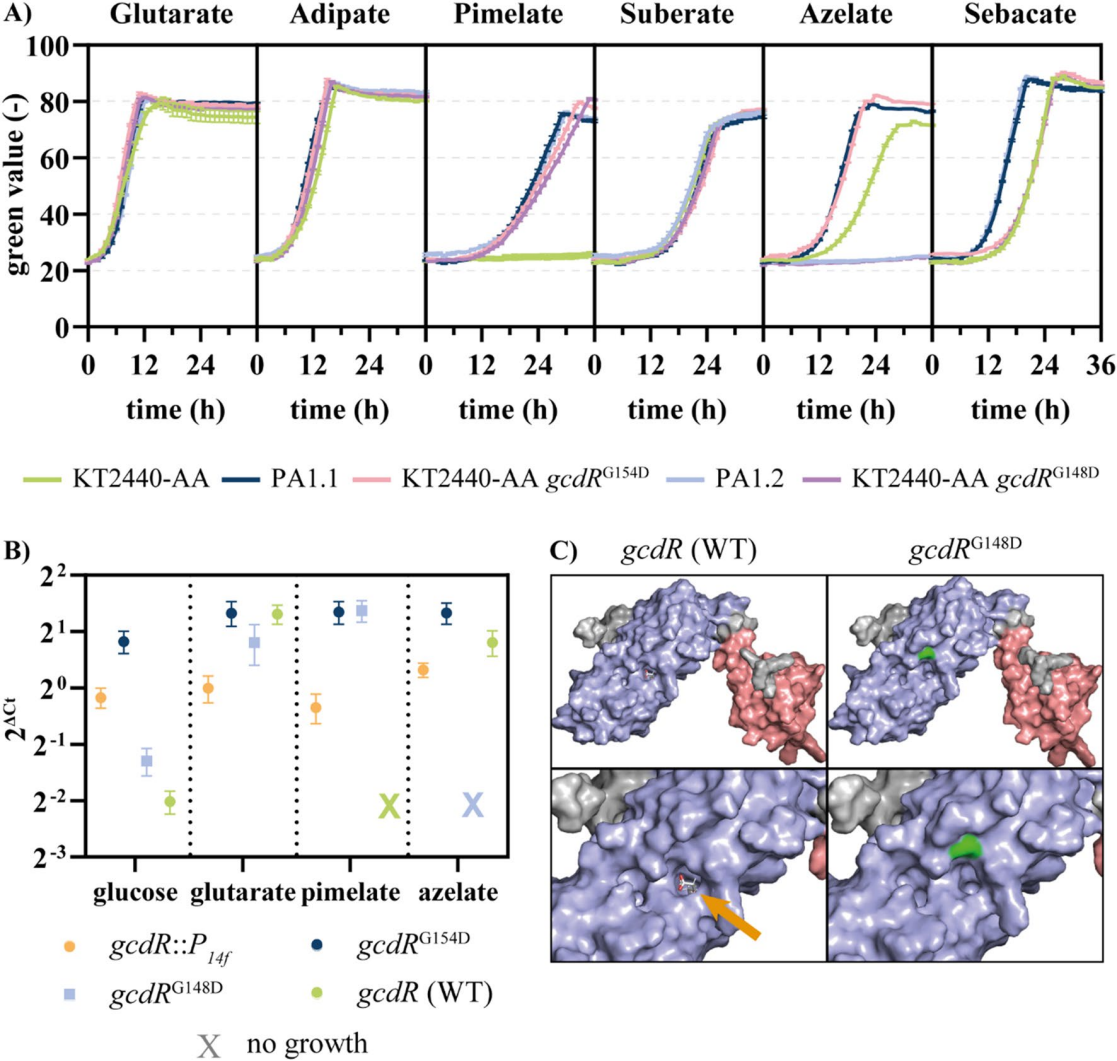
Characterization of growth of engineered and evolved strains of *P. putida *on dicarboxylic acids of varying chain lengths. **A** All strains were cultured in MSM containing the specified carbon source, that are C-mol equivalent to 30 mM adipate. The growth was monitored using a Growth Profiler. Error bars indicate the standard error of the mean (*n* = 3). **B** Relative expression levels of *gcdH* in cells of *P. putida* with wild type or mutated versions of the regulator GcdR on different C-sources were determined by RT-qPCR. The Ct values were normalized to the Ct of *rpoD*. Standard errors of the means were calculated using three technical replicates of two biological replicates. Expression levels in cells that did not grow on certain substrates were set equal to unexpressed values and are indicated with “X”. **C** Three-dimensional structures were predicted with ColabFold and visualized with PyMOL. Docking of glutaric acid in the wild type regulator was calculated using YASARA (orange arrow). The mutated amino acid (D148) is marked in green. The blue surface color indicates the effector binding domain and the red surface color indicates the DNA binding domain. The visualization of the mutant gcdR^G154D^ is shown in Fig. S6

To comprehend the effects of the G148D and G154D mutations on GcdR, RT-qPCR experiments were performed to analyze expressions levels of *gcdH* in the *gcdR* mutants on different ucl-DCA and on glucose (Fig. 3). In the GcdR^G154D^ mutant, the expression levels of *gcdH* are the same on all substrates. Hence, this mutation likely led to a strong constitutive activation, at a level similar to the wild type induced by glutarate. In contrast, in the GcdR^G148D^ mutant, expression levels of *gcdH* are much higher on glutarate and pimelate than on glucose or azelate, indicating that this mutant is induced by both ucl-DCA, in contrast to the wild type regulator which is only induced by glutarate. This indicates that the G148D mutation increased the spectrum of possible ligands of GcdR. ColabFold protein structure simulations and YASARA docking studies [41, 42] indicate a structural impact of G148D and G154D on the effector binding pocket of GcdR (Fig. 3, Fig. S6). The G148D substitution is more distal from the pocket, which appears to be larger compared to the wild type. The G154D mutation is closer to the pocket, where the negatively charged aspartic acid might lead to a conformational change that is not easily modelled by this in silico method. This would support the RT-qPCR results, although these are only simulations that need further confirmation. The expression levels of *gcdH* with the wild type regulator induced by glutarate, the G148D mutant induced by glutarate or azelate, and the constitutive G154D mutant, are similarly high, significantly exceeding that of the constitutive *P*_*14f*_ promoter exchange strain. This supports that the slow growth of the latter strain was caused by the relatively weak expression driven by the promoter exchange.

With this knowledge, the strains containing the SNVs were compared to *P. putida* KT2440-AA in terms of growth on different mcl-DCA. This confirmed the improvement of growth of the GcdR^G154D^ strain on ucl-DCA compared to the starting strain (Fig. 3). The most conspicuous difference can be seen on azelate. The parent strain with wild type *gcdR* grew reasonably well on this C_9_-DCA, possibly as a result of the two acetyl-CoA released from β-oxidation of this longer chain length. However, growth on azelate was enhanced by the G154D mutation but inhibited by G148D. This indicated that the G148D mutation altered the effector binding pocket such that pimelate causes induction, while azelate causes repression.

### Enabling growth on ucl 1,7-heptanediol

**Fig. 4:**
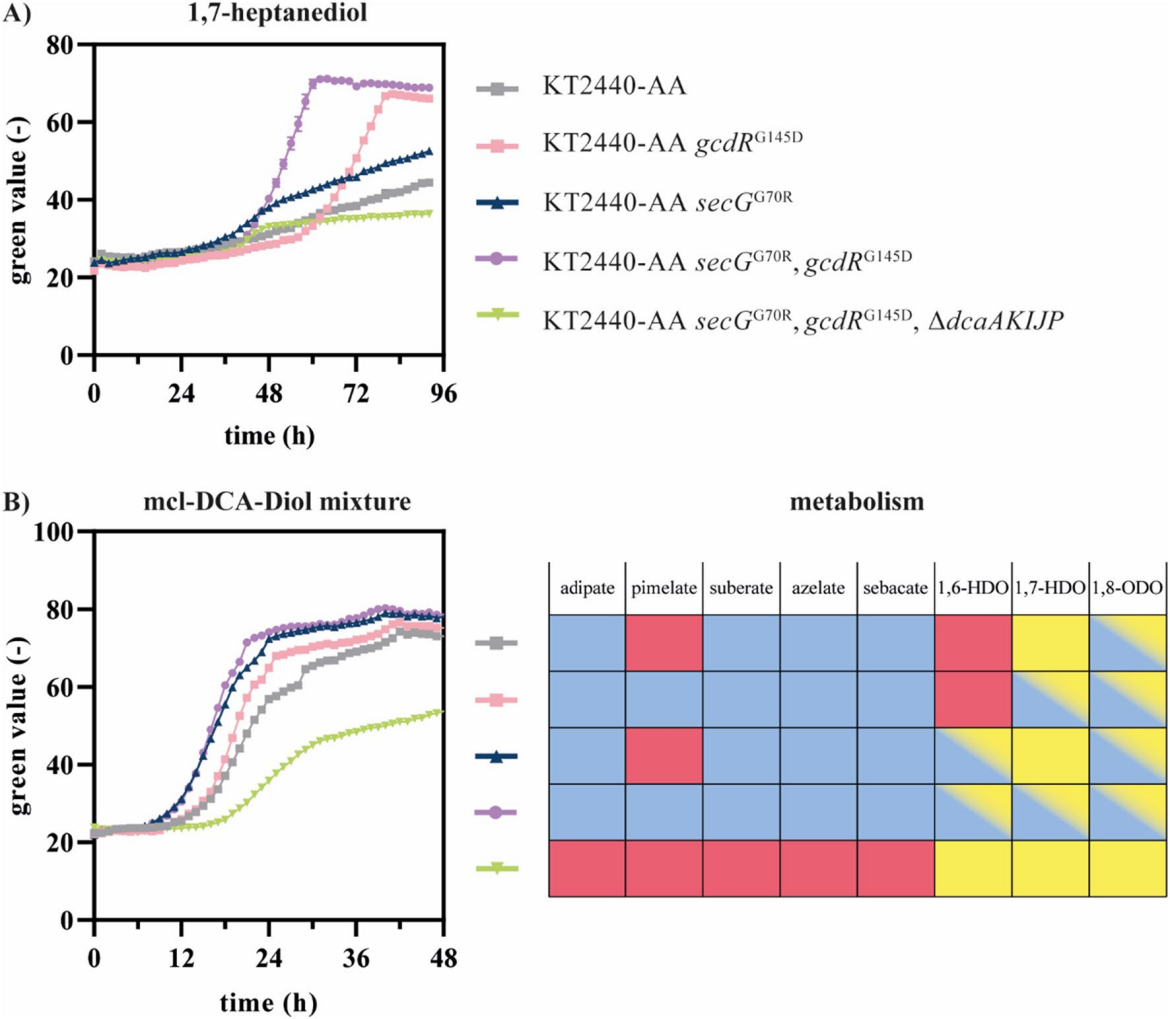
Growth of KT2440-AA strains on 1,7-heptanediol and on a mcl-DCA -diol mixture. All strains were cultivated in MSM containing 25.7 mM 1,7-heptanediol (**A**) or a mixture consisted of adipate, pimelate, suberate, azelate, sebacate, 1,6-hexanediol (HDO), 1,7-heptanediol, and 1,8-octanediol (ODO) with concentrations of 3 mM each (**B**). Red indicates the inability of the strain to metabolize the substrate. Yellow indicates metabolism *via* the HA-CoA-activating pathway, whereas blue indicates that the substrate was metabolized via the DCA-CoA-activating pathway. Potential activity of both pathways is indicated as color gradient. All mutations shown are in the KT2440-AA strain. Error bars indicate the standard error of the mean (*n* = 3)

*P. putida* KT2440-AA *secG*^G70R^ metabolized diols of even chain-length via the DCA-CoA-activating pathway, whereas the ucl 1,7-heptanediol was poorly metabolized by this strain due to its inability to utilize pimelate. Since introducing the *gcdR*^G154D^ mutation into *P. putida* KT2440-AA enabled metabolism of pimelate as sole carbon source, it was introduced into *P. putida* KT2440-AA *secG*^G70R^. Indeed, the resulting *secG*^G70R^, *gcdR*^G154D^ mutant metabolized 1,7-heptanediol and deletion of the *dcaAKIJP* cluster confirmed its metabolism *via* the DCA-CoA-activating pathway (Fig. 4). The *gcdR*^G154D^ mutation was also introduced into the wild type-based *ttgG*^∆4 bp^, PP_2046^A257T^, PP_2790^A220V^ mutant that metabolized HDO and ODO via the HA-CoA-activating pathway. However, the resulting mutant was not able to metabolize 1,7-heptanediol (data not shown). This highlights the DCA-CoA-activating pathway as most suitable pathway for funneling aliphatic even and uneven mcl-DCA and -diols into the central metabolism of the engineered *P. putida* KT2440-AA *secG*^G70R^, *gcdR*^G154D^. A mixture consisting of C_6_-C_10_ DCA and C_6_-C_8_ diols was fully consumed by this strain confirming the successful funneling of all substrates from a complex mixture into the central metabolism (Fig. 4).

### Towards bio-upcycling of complex aliphatic mixtures

**Fig. 5:**
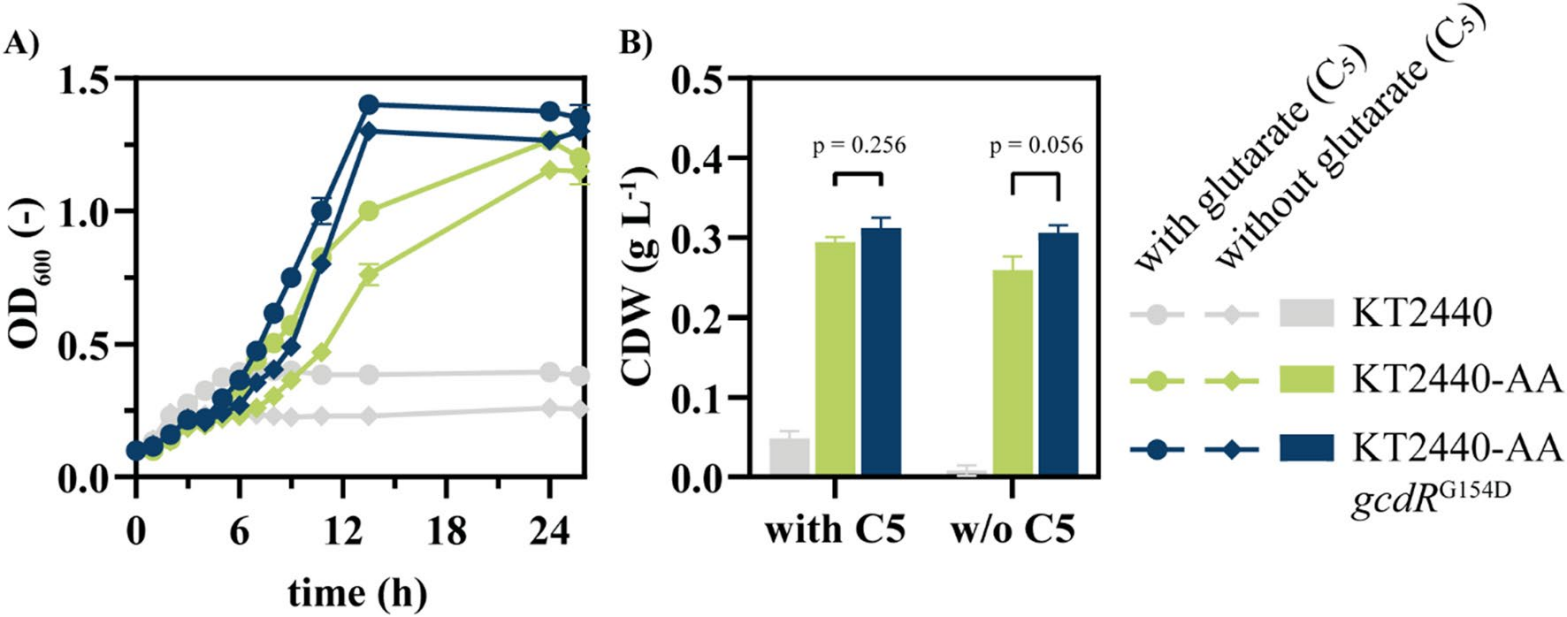
Growth of strains on a mixture of various (mcl)-dicarboxylic acids. All strains were cultivated in three-fold buffered MSM containing 1 mM of each DCA (C_4_-C_10_) with and without glutarate to compare the influence of inducer. For offline growth measurements, samples were taken at several time points (**A**). Final consumption of all monomers was confirmed by HPLC (data not shown). After 26 h sample were taken for final cell dry weight determination (**B**). Error bars indicate the standard error of the mean (*n* = 2)

Although growth on single monomers is useful to elucidate the genetic and biochemical basis of mcl-DCA metabolism, for bio-upcycling purposes it is necessary to metabolize mixtures of complex plastic hydrolysates. For example, auto-oxidation of high-density polyethylene (HDPE) yields a mixture of C_4_-C_22_ dicarboxylic acids [8]. This mixture was successfully degraded by Sullivan et al. by strain AW162 comparable to *P. putida* KT24440-AA described above [8]. Strain AW162 lacks the mutations in *gcdR* and is not able to grow on pimelate as sole carbon source [8]. However, AW162 was able to metabolize ucl-DCA in a mixture, likely due to the presence of glutarate to induce *gcdH*-PP_0159. We hypothesized that the relatively low concentration of glutarate might lead to sub-optimal expression that might be ameliorated by the *gcdR* mutation. Indeed, comparison of growth of our reverse engineered strain with and without *gcdR*^G154D^ on a mixture of C_4_-C_10_ DCA reveals a much better growth for the strain harbouring the mutation (Fig. 5). This was the case for mixtures with and without glutarate.

**Fig. 6:**
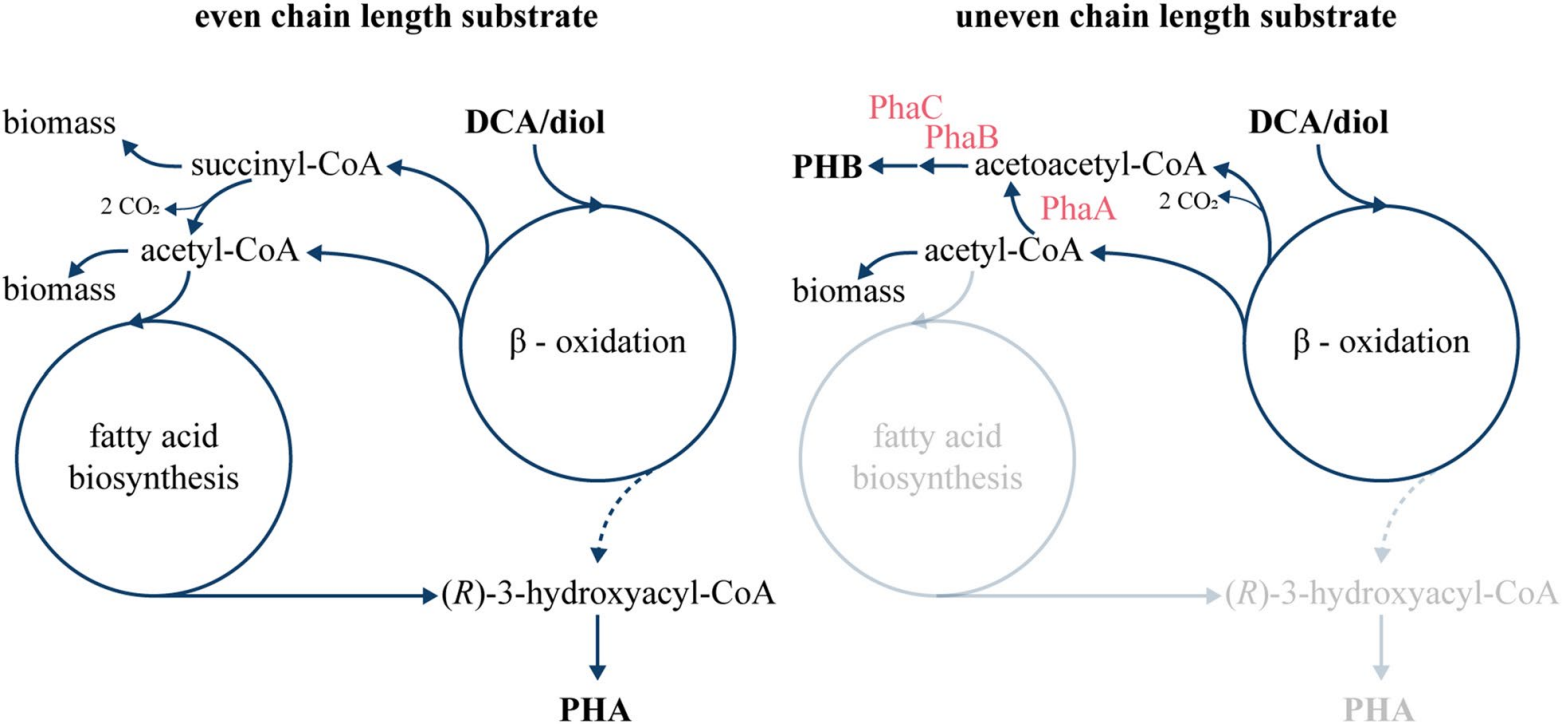
Comparison between PHA and PHB synthesis from even- and uneven-chain aliphatic diols and dicarboxylates. The heterologous enzymes from *C. necator* responsible for PHB production are shown in red

The successful funneling of DCA and diols of even and uneven chain lengths into the central metabolism of our engineered *P. putida* KT2440-AA *secG*^G70R^, *gcdR*^G154D^ paves the way for investigating their bio-upcycling. As target product, polyhydroxyalkanoates (PHA) were selected that are biodegradable polyesters with increasing industrial applications [21, 43]. In nitrogen-limited media, *P. putida* KT2440 natively produces mcl-PHA providing (*R*)-3-hydroxyacyl-CoA, the primary precursor, via two pathways [36]. By this, related substrates such as fatty acids are converted to (*R*)-3-hydroxyacyl-CoA via β-oxidation, whereas unrelated substrates such as glucose are funneled via malonyl-CoA into fatty acid de novo synthesis resulting in the production of the precursor. To test if the engineered *P. putida* KT2440-AA *secG*^G70R^, *gcdR*^G154D^ is able to produce mcl-PHA from mcl-DCA and -diols, the strain was cultivated in nitrogen-limited medium with a C:N ratio of 30:1 using substrate concentrations that are C-mol equivalent to 30 mM adipate. Although all mcl-DCA are metabolized via the *dcaAKIJP*-encoded β-oxidation, PHA production was clearly dependent on the chain-length of the substrate (Table 1). Using adipate as substrate, mcl-PHA were produced to 15.7 ± 1.0% of the cell dry weight (CDW) with 3-hydroxydecanoic acid as the dominant monomer (57.8 ± 3.2%). In contrast to this, only 3.3 ± 0.1% mcl-PHA were produced from pimelate and less than 1% mcl-PHA were produced from suberate, azelate, and sebacate. The same trend was observed when different mcl-diols were tested for mcl-PHA production. In total, 10.0 ± 0.6% mcl-PHA were produced from HDO and 4.3 ± 0.1% from 1,7-heptanediol. As observed for suberate, mcl-PHA production from ODO was below 1%. Although the relative monomer composition of adipate and pimelate compared to HDO and 1,7-heptanediol was similar, the total amount of mcl-PHA was higher when the mcl-DCA were used as substrates (Table 1). This can be explained by the presence of the HA-CoA-activating pathway for mcl-diol metabolism in the ∆*dcaAKIJP* mutants. Hence, less carbon was likely funneled from the diols into the DCA-CoA-activating pathway yielding less favourable precursors for mcl-PHA production. When a mock hydrolysate consisting of C_6_-C_10_-DCA and C_6_-C_10_-diols with 5 mM each was used as substrate, 0.3% ± 0.0 mcl-PHA were produced. This low yield can be explained by the relative high amount of C_8_-C_10_ substrates that were identified to be barely suitable for mcl-PHA production. Our results fit into the observations of Sullivan et al. that reported a yield of 11.8 ± 2.9% mcl-PHA from a mixture containing benzoate, acetate, and C_4_-C_17_-mcl-DCA [8]. When a polystyrene hydrolysate was tested, only 0.8% ± 0.2% mcl-PHA were produced indicating that only fractions of complex mixtures can be used to produce PHA. In contrast to C_8_-C_10_-fatty acids that are well-suited for mcl-PHA production in *P. putida* KT2440 [20], C_8_-C_10_-DCA were not appropriate for mcl-PHA production. This likely results from the fact that mcl-DCA such as adipate and pimelate are metabolized *via* β-oxidation, but unlike fatty acids they are not directly used as PHA precursors. Rather they are broken down to acetyl-CoA, and then shunted back into fatty acid *de novo* synthesis, which is linked back to β-oxidation through the action of PhaG. Possibly, the longer-chain DCA, which match the typical PHA monomer chain length, induce components of β-oxidation that interfere with PHA synthesis by degrading the hydroxyacyl-CoA precursor. Moreover, the ratio between succinyl-CoA and acetyl-CoA increases with increasing chain-length using C-molar equivalent concentrations of the substrate. The changing ratio might influence PHA production (Fig. 6). This could also be an explanation for the variation in CDWs when different substrates are metabolized (Table 1).

**Table 1:** Production of mcl-PHA by engineered *P. putida* KT2440-AA secG^G70R^, gcdR^G154D^from different substrates

Substrate	CDW (g L^−1^)	PHA (%)	C_6_ (%)	C_8_ (%)	C_10_ (%)	C_12_ (%)
adipate	0.62 ± 0.05	15.7 ± 1.05	14.3 ± 3.2	24.1 ± 0.2	57.8 ± 3.2	3.8 ± 0.2
pimelate	0.62 ± 0.02	4.3 ± 0.12	14.2 ± 0.2	32.3 ± 0.3	47.9 ± 0.2	5.6 ± 0.0
suberate	0.47 ± 0.00	0.4 ± 0.01	n. d.	5.0 ± 1.1	48.4 ± 1.6	46.6 ± 3.4
azelate	0.47 ± 0.01	0.4 ± 0.02	n. d.	n. d.	48.8 ± 3.4	51.2 ± 3.4
sebacate	0.52 ± 0.03	0.6 ± 0.01	n. d.	9.9 ± 0.2	55.8 ± 0.7	34.3 ± 0.4
1,6-hexanediol	0.57 ± 0.03	10.0 ± 0.6	10.0 ± 0.5	33.4 ± 0.2	52.9 ± 0.1	3.8 ± 0.2
1,7-heptanediol	0.57 ± 0.01	3.4 ± 0.01	12.8 ± 0.7	30.0 ± 0.0	49.7 ± 0.2	7.5 ± 0.5
1,8-octanediol	0.43 ± 0.01	0.5 ± 0.00	4.7 ± 0.5	7.2 ± 0.1	49.1 ± 2.8	39.0 ± 2.0
mock hydrolysate	0.89 ± 0.02	0.3 ± 0.01	n. d.	n. d.	60.9 ± 1.1	39.1 ± 1.1

The CDW, PHA content, and relative monomer composition of mcl-PHA are shown. The strain was cultivated in MSM supplemented with C-mol equimolar concentrations to 30 mM of adipate using a C:N ratio of 30:1. The mock hydrolysate consisted of 5 mM of each individual substrate. Error values are calculated as standard deviations (*n* = 2). Exemplary GC chromatograms are shown in Fig. S7

To avoid the abovementioned hypothesized conflict, we investigated whether PHB might be a favoured product for the bio-upcycling of the described substrates. This short-chain polymer is produced from acetoacetyl-CoA, which is converted to (*R*)-3-hydroxybutyryl-CoA as substrate for PHB synthesis. To produce PHB in *P. putida* KT2440, the ability to produce mcl-PHA was abolished by deleting the PP_5003-6 gene cluster including the PHA (de-)polymerases. Since *P. putida* KT2440 is not a natural PHB producer, a PHB biosynthesis pathway from *C. necator* H16 was expressed in the KT2440-AA *secG*^G70R^*gcdR*^G154D^ mutant. The synthetic pathway comprises *phaCAB*, encoding (i) PhaA, a thiolase that condenses 2 acetyl- CoA moieties into acetoacetyl-CoA, (ii) PhaB, a reductase that converts acetoacetyl-CoA into (*R*)-3-hydroxybutyryl-CoA and (iii) PhaC, a short-chain-length (scl)-PHA synthase that polymerizes 3-hydroxybutyryl-CoA monomers (C_4_) to yield PHB. We constructed a synthetic operon with these genes under the transcriptional control of the ChnR/P_*chnB*_ expression system, inducible by cyclohexanone [44], and a synthetic ribosome binding site (5’-AGG AGG AAA AAC AT-3’) upstream of each gene. This construct was assembled in the pSEVA631 vector by USER cloning, resulting in plasmid pS6311·PHB.

Heterologous expression of the *phaCAB* cluster indeed enabled production of PHB by *P. putida* KT2440-AA *secG*^G70R^, *gcdR*^G154D^ carrying pS6311*·*PHB under nitrogen-sufficient conditions (Table 2). In contrast to native mcl-PHA production, uneven substrates such as pimelate (15.14 ± 0.05%) and 1,7-heptanediol (21.86 ± 2.77%) were preferred for PHB accumulation. This can be explained by the formation of acetoacetyl-CoA as intermediate that is directly used for PHB synthesis. Hence less carbon is available for biomass formation yielding lower CDWs (Table 2). When azelate was used as substrate, less PHB (6.53 ± 0.92%) was produced compared to pimelate likely caused by the formation of an additional molecule of acetyl-CoA that was used for the production of biomass as indicated by the CDW.

Although mcl-PHA were produced from adipate, this substrate as well as suberate and sebacate were not suited for the production of PHB as acetyl-CoA was likely used for biomass formation (Table 2). Moreover, HDO and ODO also resulted in lower amounts of PHB. In all, these results indicate that the produced PHB was mainly derived from acetoacetyl-CoA as intermediate of ucl-DCA or -diol metabolism. Consequently, our results indicate that acetyl-CoA from substrates with even chain length are predominantly utilized for the production of biomass rather than converted to acetoacetyl-CoA for PHB synthesis. To enable efficient upcycling of substrates with even and uneven chain lengths present in mixed hydrolysates, future studies could investigate the combination of scl- with mcl-PHA synthesis pathways in a single strain. By this both mcl- and ucl-substrates can be converted to scl-co-mcl-PHA that features enhanced physical properties compared to homopolymeric PHAs [45].

**Table 2:** Production of PHB by engineered *P. putida *KT2440-AA *secG*^G70R^, *gcdR*^*G1*54D ^carrying plasmid pS6311·PHB from different substrates

Substrate	CDW (g L^−1^)	PHB (%)
Adipate	1.52 ± 0.05	1.16 ± 0.06
Pimelate	0.23 ± 0.01	15.14 ± 0.05
Suberate	1.52 ± 0.04	1.56 ± 0.19
Azelate	1.47 ± 0.01	6.53 ± 0.92
Sebacate	1.52 ± 0.02	2.35 ± 0.07
1,6-Hexanediol	1.29 ± 0.14	3.59 ± 0.01
1,7-Heptanediol	0.26 ± 0.01	21.86 ± 2.77
1,8-Octanediol	0.56 ± 0.01	2.20 ± 0.76
Mock hydrolysate	1.16 ± 0.01	3.59 ± 0.01

The CDW and PHB content are shown. The strain was cultivated in MSM supplemented with C-equimolar concentrations of pure substrates and 1 mM cyclohexanone as inducer for *phaCAB* expression. The strain was cultivated in MSM supplemented with C-mol equimolar concentrations to 30 mM of adipate using a C:N ratio of 30:1. The mock hydrolysate consisted of 5 mM of each individual substrate. Error values are calculated as standard deviations (*n* = 2). Exemplary GC chromatograms are shown in Fig. S7

## Conclusions

Bio-upcycling of complex monomer mixtures, either from a single polymer or from mixed plastic waste streams, is a promising approach for the establishment of a circular economy. In order to achieve an efficient bio-upcycling approach, the funneling of different monomers into the value-added products is crucial. We successfully engineered the combined degradation of diols and dicarboxylic acids in a single platform strain of *P. putida* KT2440. Using this strain, we demonstrated the conversion of monomer mixtures into different PHA, which are of increasing interest in the polymer industry. Although PHA yields are currently relatively low (± 0.03 g_PHA_ g_substrate_^−1^), our study provides fundamental insights into the different metabolic pathways available for aliphatic α,ω-functionalized molecules and how their central metabolic products affect product formation. This leads the path for future PHA yield optimizations and increased the range of potential substrates for PHA production.

### Supplementary information

**Additional file 1: Figure S1.** Adaptive laboratory evolution of *P. putida* KT2440 wild type (A) and KT2440-AA (B) on 1,6-hexanediol. **Figure S2.** Metabolic pathways of aliphatic diols in engineered *P. putida* KT2440. **Figure S3.** Growth of *P. putida* KT2440-AA mutants on HDO. **Figure S4.** Growth of *P. putida* KT2440-AA mutants on 6-hydroxyhexanoate. **Figure S5.** Growth of *P. putida* KT2440-AA mutants on glutarate. Strains were cultivated in MSM supplemented with 36 mM glutarate as sole carbon source. **Figure S6.** Three-dimensional structures of GcdR predicted with ColabFold and visualized with PyMOL. **Figure S7.** Exemplary gas chromatography chromatograms of polyhydroxyalkanoates (A) and polyhdroxybutyrate (B). **Table S1.** Strains used in this work. **Table S2.** Oligonucleotides used in this work. **Table S3.** Plasmids used in this work.

## Data Availability

All data generated or analysed during the study are included in this published article and its additional file informations. Sequencing data are deposited in the NCBI Sequence Read Archive under BioProject number PRJNA987418.
