# Supplementary material for: Bio-upcycling of even and uneven medium-chain-length diols and dicarboxylates to polyhydroxyalkanoates using engineered Pseudomonas putida
Source: Microb Cell Fact. 2024 Feb 16;23:54. doi: 10.1186/s12934-024-02310-7 (PMC10870600; doi:10.1186/s12934-024-02310-7)
Supplement: Supplementary file 1 — Additional file 1: Figure S1. Adaptive laboratory evolution of P. putida KT2440 wild type (A) and KT2440-AA (B) on 1,6-hexanediol. Figure S2. Metabolic pathways of aliphatic diols in engineered P. putida KT2440. Figure S3. Growth of P. putida KT2440-AA mutants on HDO. Figure S4. Growth of P. putida KT2440-AA mutants on 6-hydroxyhexanoate. Figure S5. Growth of P. putida KT2440-AA mutants on glutarate. Strains were cultivated in MSM supplemented with 36 mM glutarate as sole carbon source. Figure S6. Three-dimensional structures of GcdR predicted with ColabFold and visualized with PyMOL. Figure S7. Exemplary gas chromatography chromatograms of polyhydroxyalkanoates (A) and polyhdroxybutyrate (B). Table S1. Strains used in this work. Table S2. Oligonucleotides used in this work. Table S3. Plasmids used in this work. [file 12934_2024_2310_MOESM1_ESM.pdf]

Supplements for:

Bio-upcycling of even and uneven medium-chain-length diols and dicarboxylates to polyhydroxyalkanoates using engineered *Pseudomonas putida*

Yannic S. Ackermann[a†], Jan de Witt [a†], Mariela P. Mezzina[b], Christoph Schroth[a], Tino Polen[a], Pablo I. Nikel[b], Benedikt Wynands[a], Nick Wierckx[a*]

[†] YSA and JdW contributed equally to this study.

[a] Institute of Bio- and Geosciences IBG-1: Biotechnology, Forschungszentrum Jülich, Jülich, Germany

[b] The Novo Nordisk Foundation Center for Biosustainability, Technical University of Denmark, Kongens Lyngby, Denmark

*to whom correspondence should be addressed:

Prof. Dr. Nick Wierckx

Phone: +49 246161 85247

Fax: +49 246161 2710

email: n.wierckx@fz-juelich.de

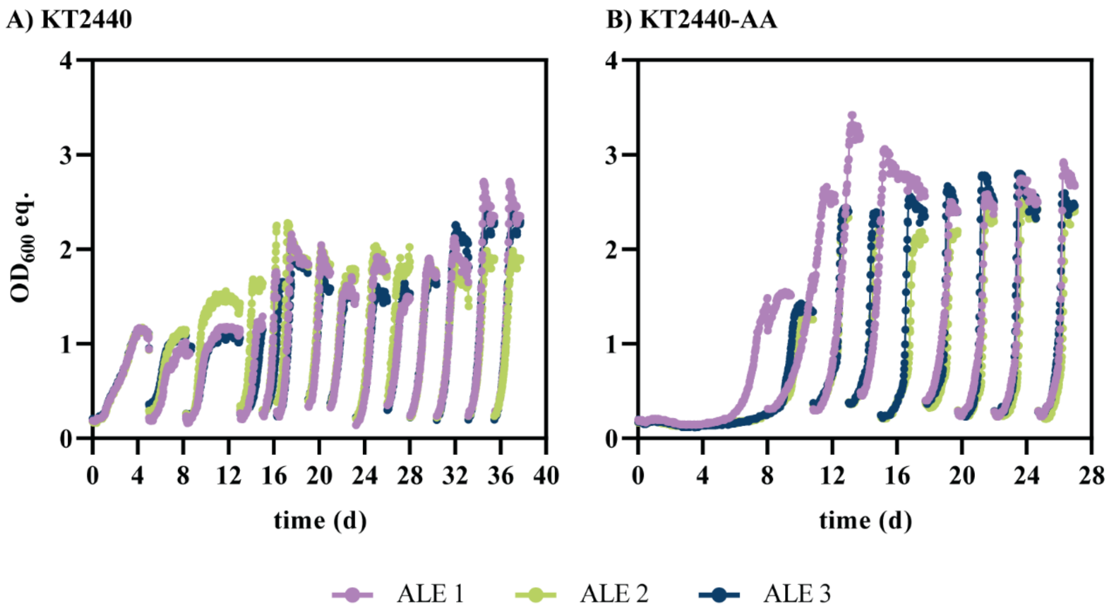

Figure S1. Adaptive laboratory evolution of *P. putida* KT2440 wild type (A) and KT2440-AA (B) on 1,6-hexanediol. ALE was performed by iterative inoculation after the stationary phase was reached. Since *P. putida* KT2440 did not grow with HDO as sole carbon source, 15 mM HDO and 15 mM BDO were used for the first two stages of ALE to enable growth. This concentration was shifted to 20 mM HDO and 10 mM BDO (stages 3-5), and to 30 mM HDO (stages 6-14). *P. putida* KT2440-AA could grow with 30 mM HDO.

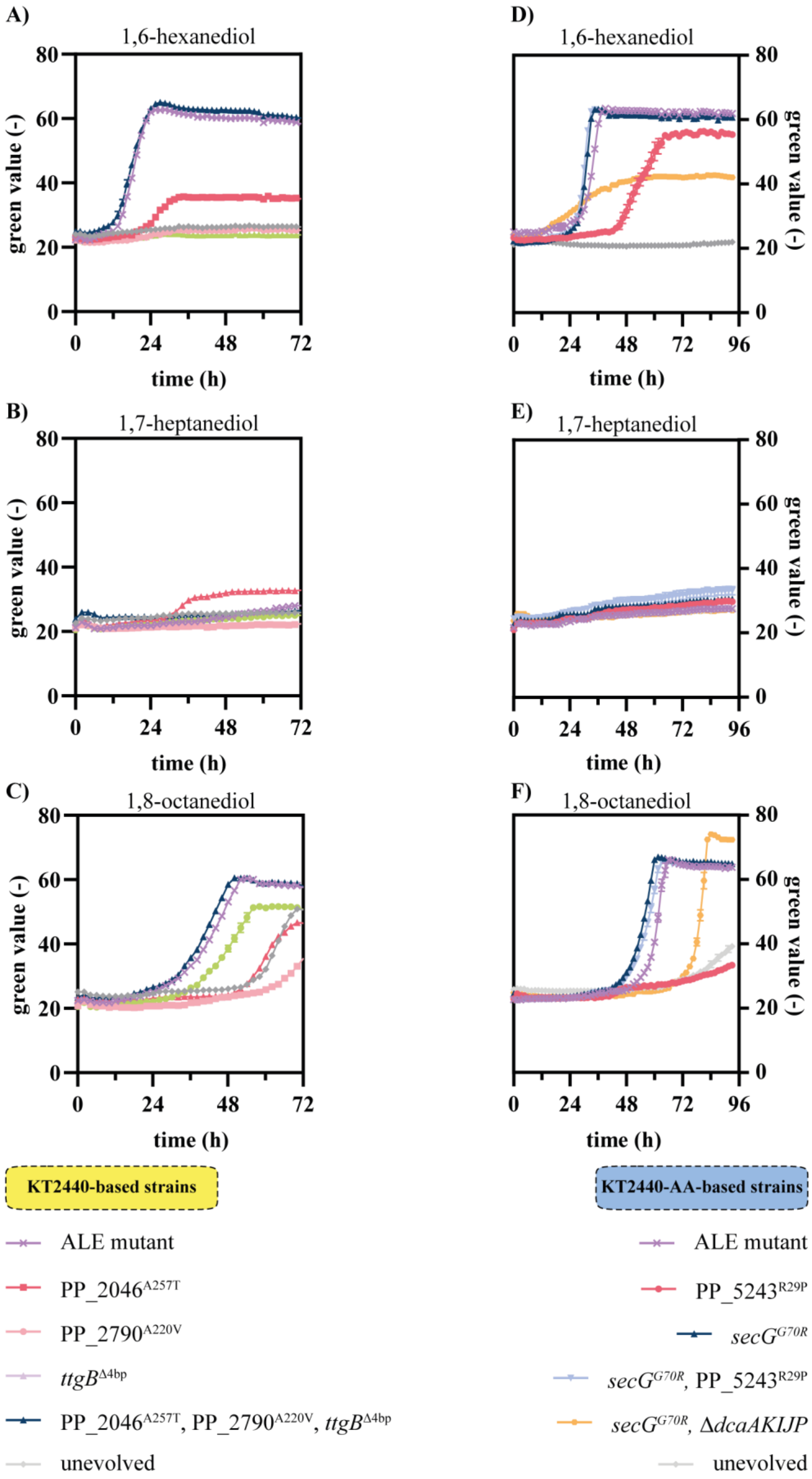
A)
1,6-hexanediol
green value (-)
time (h)
D)
1,6-hexanediol
green value (-)
time (h)
B)
1,7-heptanediol
green value (-)
time (h)
E)
1,7-heptanediol
green value (-)
time (h)
C)
1,8-octanediol
green value (-)
time (h)
F)
1,8-octanediol
green value (-)
time (h)
KT2440-based strains
ALE mutant
PP_2046A257T
PP_2790A220V
ttgBΔ4bp
PP_2046A257T, PP_2790A220V, ttgBΔ4bp
unevolved
KT2440-AA-based strains
ALE mutant
PP_5243R29P
secGG70R
secGG70R, PP_5243R29P
secGG70R, ΔdcaAKIJP
unevolved

Figure S2. Metabolic pathways of aliphatic diols in engineered *P. putida* KT2440. Extension of figure 1. *P. putida* KT2440 wild type-based strains (A-C) and *P. putida* KT2440-AA-based strains (D-F) were cultivated in mineral salts medium (MSM) supplemented with 1,6-hexanediol, 1,7-heptanediol, or 1,8-octanediol in concentrations that are C-mol equivalent to 30 mM 1,6-hexanediol. Growth was monitored using a Growth Profiler. Error bars indicate the standard error of the mean (n=3).

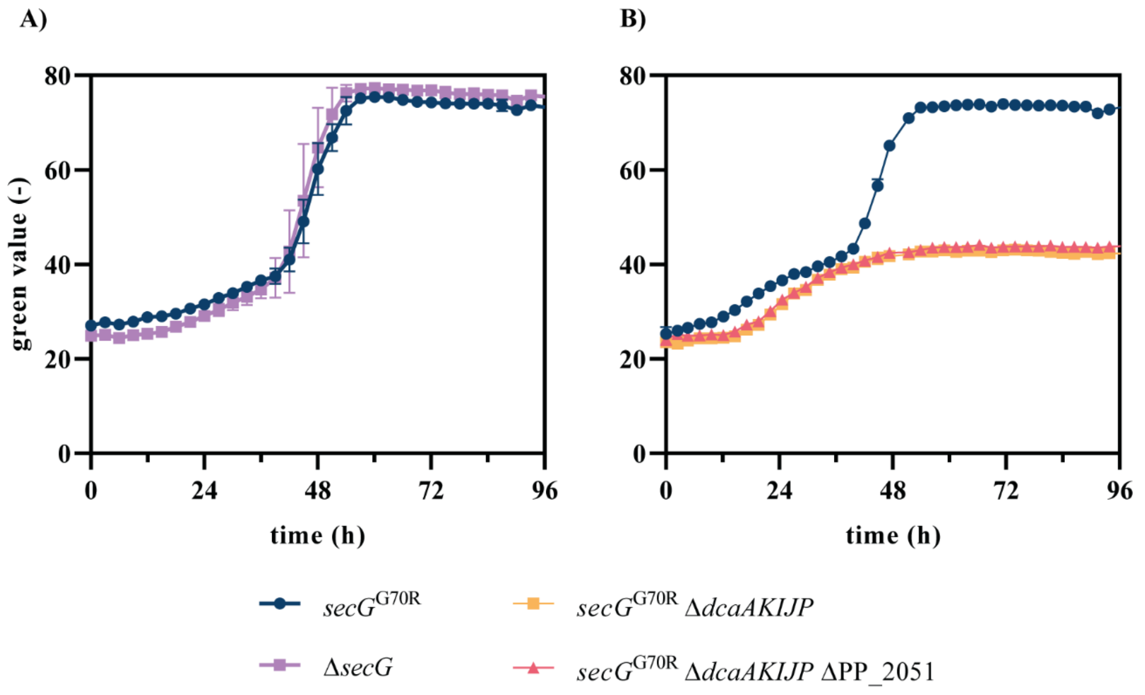

Figure S3. Growth of *P. putida* KT2440-AA mutants on HDO. Strains were cultivated in MSM supplemented with 15 mM of HDO as sole carbon source. (A) Effect of *secG*^{G70R} mutation on HDO metabolism. (B) Effect of Δ*dcaAKIJP* and ΔPP_2051.

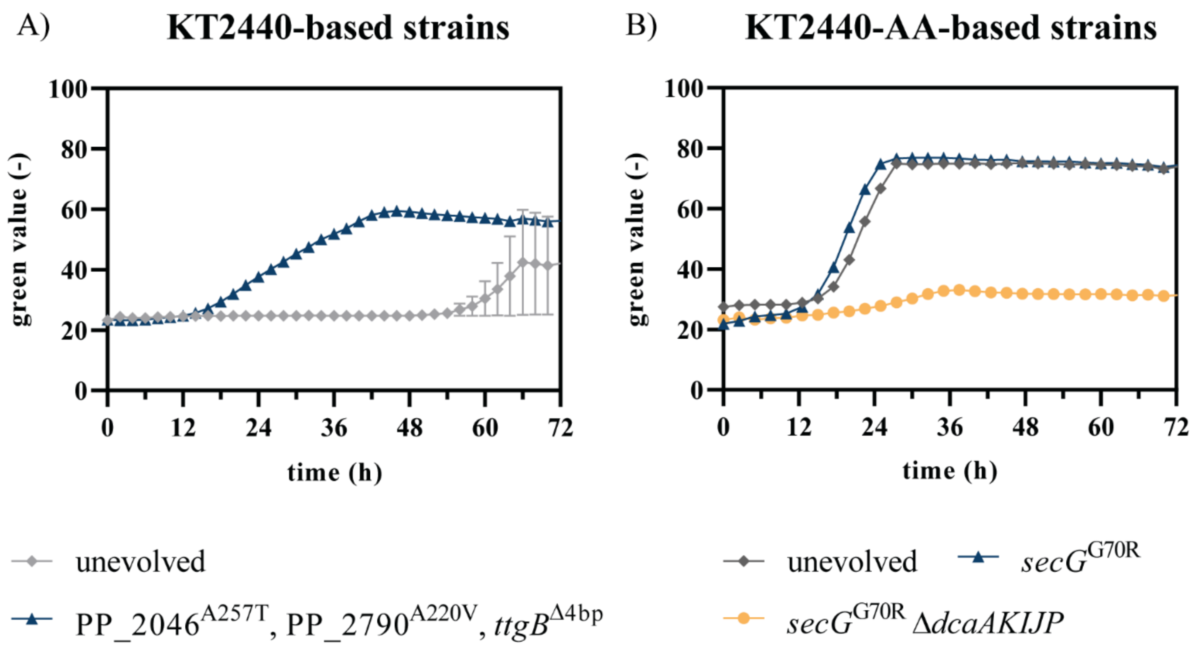

Figure S4. Growth of *P. putida* KT2440-AA mutants on 6-hydroxyhexanoate. Strains were cultivated in MSM supplemented with 15 mM of 6-hydroxyhexanoate as sole carbon source.

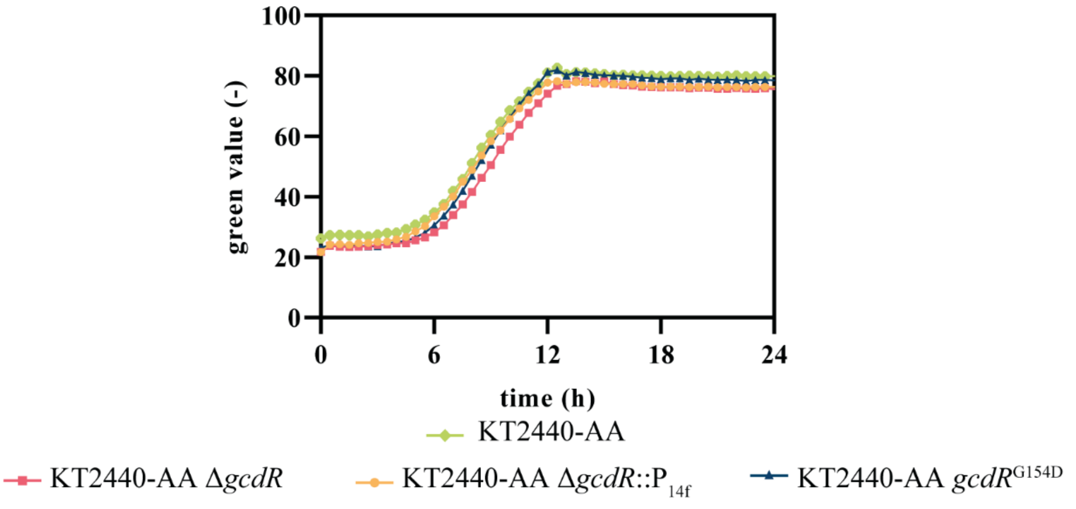

Figure S5. Growth of P. putida KT2440-AA mutants on glutarate. Strains were cultivated in MSM supplemented with 36 mM glutarate as sole carbon source.

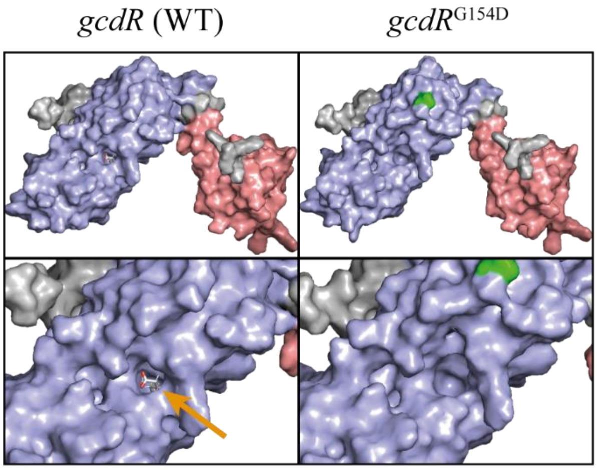

Figure S6. Three-dimensional structures of GcdR predicted with ColabFold and visualized with PyMOL. Docking of glutaric acid was calculated using YASARA (orange arrow). Mutated amino acid (D154) is marked in green. The blue surface color indicates the effector binding domain and the red surface color indicates the DNA binding domain.

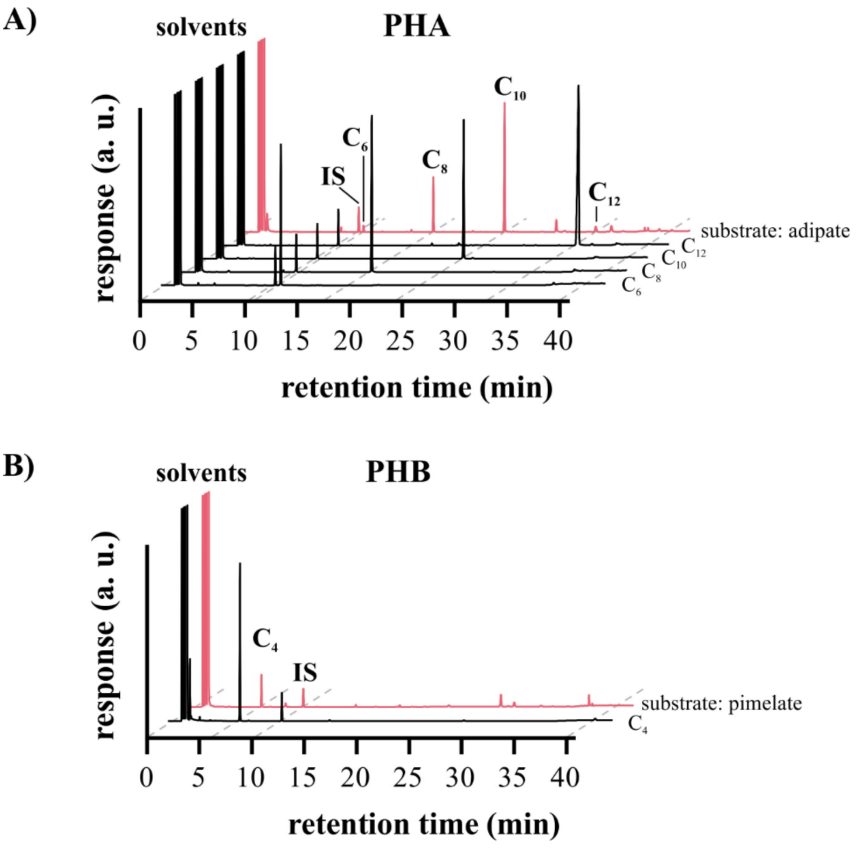

Figure S7. Exemplary gas chromatography chromatograms of polyhydroxyalkanoates (A) and polyhdroxybutyrate (B). Methyl esters of 3-hydroxyacids (C_4-C_{12}) were quantified using analytical standards (black lines). As internal standard (IS) methyl benzoate was used. PHA production from adipate (A) and PHB production from pimelate (B) are shown in red. For better visibility, the y-axis was capped due to high responses of chloroform and methanol.

Table S1: Strains used in this work.

P. putida **strain**	**Description**	**Reference**
**KT2440**	Strain derived from *P. putida* mt-2 cured of the pWW0 plasmid	Bagdasarian et al. (1981)
**A12.1p**	Evolved KT2440 strain bearing the evolved plasmid pBNT-*dcaAKIJJP*	Ackermann et al. (2021)
**A12.1ge**	A12.1 after genomic integration of *attTn7*::P_{14e}-*dcaAKIJP* and removal of the resistance marker	Ackermann et al. (2021)
**KT2440ge ΔP_{paaF}-*paaYX*::P_{14g} Δ*psrA* (KT2440-AA)**	Exchange of the natural promoter P_{paaF} for the synthetic P_{14g} promoter together with knockout of *paaYX,* knockout of *psrA*	Ackermann et al. (2021)
**PA1.1**	Evolved A12.1ge strain for growth on pimelate with *gcdR*^{G154D}	This work MiCat #2230
**PA1.2**	Evolved A12.1ge strain for growth on pimelate with *gcdR*^{G148D}	This work MiCat #2231
**KT2440-AA Δ*gcdR***	Knockout of *gcdR*	This work MiCat #1447
**KT2440-AA Δ*gcdR*::P_{14f}**	Exchange of the regulator gene *gcdR* for the synthetic P_{14f} promoter	This work MiCat #1446
**KT2440-AA *gcdR*^{G154D}**	*gcdR*^{G154D}	This work MiCat #1459
**KT2440-AA *gcdR*^{G148D}**	*gcdR*^{G148D}	This work MiCat #1454
**KT2440 ALE HDO**	Evolved KT2440 wild type on 1,6-hexanediol	This work MiCat #1560
**KT2440 PP_2046^{A257T}**	Partly reverse engineered, PP_2046^{A257T}	This study MiCat #1675
**KT2440 PP_2790^{A220V}**	Partly reverse engineered, PP_2790^{A220V}	This study MiCat #1712
**KT2440 *ttgB*$^{\Delta 4bp}$**	Partly reverse engineered, PP_1385$^{\Delta 4bp}$	This study MiCat #1713
**KT2440 PP_2046^{A257T}, PP_2790^{A220V}, *ttgB*$^{\Delta 4bp}$**	Fully reverse engineered strain	This study MiCat #1717
**KT2400-AA ALE HDO**	Evolved KT2440-AA on 1,6-hexanediol	This study MiCat #1558

**KT2440-AA *secG*^{G70R}**	Fully reverse engineered strain, *secG*^{G70R}	This work<br>MiCat #1678
**KT2440-AA PP_5423^{R29P}**	Partly reverse engineered, PP_5423^{R29P}	This work<br>MiCat #1677
**KT2440-AA *secG*^{G70R} PP_5423^{R29P}**	*secG*^{G70R}, PP_5423^{R29P} combined	This work<br>MiCat #1718
**KT2440-AA *secG*^{G70R} Δ*P$_{14e}$-dcaAKIJP***	Deletion of *dcaAKIJP* in KT2440-AA *secG*^{G70R}	This work<br>MiCat #1758
**KT2440-AA *secG*^{G70R} *gcdR*^{G154D}**	Final strain for (u)mcl-DCA and -diol metabolism	This work<br>MiCat #1834
**KT2440-AA *secG*^{G70R} *gcdR*^{G154D} ΔPP_5003-6, pS6311·PHB**	Strain for PHB production using pS6311·PHB, containing *phaCAB,* PHB biosynthesis pathway from *C. necator* H16	This work<br>MiCat #2174
***E. coli* strains**		
**HB101**	*F^{-} mcrB mrr hsdS20(rB^{-} mB^{-}) recA13 leuB6 ara-14 proA2 lacY1 galK2 xyl-5 mtl-1 rpsL20(*SmR*) gln V44λ^{-}*	Boyer and Roulland-dussoix, (1969)
**PIR2**	*F^{-} Δlac169 rpoS (Am) robA1 creC510 hsdR514 endA reacA1 uidA (ΔMlui)::pir*	Life technologies
**DH5α λpir**	*endA1 hsdR17 glnV44 (= supE44) thi-1 recA1 gyrA96 relA1 Φ 80dlacΔ (lacZ)M15 Δ(lacZYA-argF)U169 zdg-232::Tn10 uidA::pir^{+}*	de Lorenzo lab

Table S2: Oligonucleotides used in this work

Primer	Sequence 5´-3´	Description
JDW169	ATTCGAGCTCGGTACCCGGGAATGGCTGCTCACAGAAC	TS1 Δ*dcaAKIJP*
JDW170	TTTTAGAGAATTAAAACTGTCGCTAGAGAATTAAAG	
JDW171	ACAGTTTTAATTCTCTAAAACCAGTTGATCAACACC	TS2 Δ*dcaAKIJP*
JDW172	CAGGTCGACTCTAGAGGATCAAGCCGGTGTCGAAGCTG	
JDW208	ATTCGAGCTCGGTACCCGGGACGCTGGGCCAGGGCGAA	TS1 *ttgB*$^{\Delta 4bp}$
JDW209	GCGCCCTGGTATCGCCCTGGTGCTCTCGG	
JDW210	CCAGGGCGATACCAGGGCGCCCTGGATC	TS2 *ttgB*$^{\Delta 4bp}$
JDW211	CAGGTCGACTCTAGAGGATCGCCTGCAAACCGCCGAGC	
JDW215	ATTCGAGCTCGGTACCCGGGATGGTCATGTTGGCCAGGTC CAG	TS1 - PP_2046^{A247T} - TS2
JDW216	CAGGTCGACTCTAGAGGATCCGCTGCTGGTCCGCGTGG	
JDW218	ATTCGAGCTCGGTACCCGGGTGTGCTGCTTCGAACAGG	TS1 - PP_2790^{A222V} - TS2
JDW219	CAGGTCGACTCTAGAGGATCGCTGATCAGCCACTTGCAG	
JDW223	ATTCGAGCTCGGTACCCGGGAGCTGTACTGTCACGTCAAT ATTC	TS1 - PP_5423^{R29P} - TS2
JDW224	CAGGTCGACTCTAGAGGATCGCGGTTTGGTGAGTTTTTC	
JDW228	ATTCGAGCTCGGTACCCGGGAAGGCCTGCAACTGTTCTAG CTTGC	TS1 - *secG*^{G70R} - TS2
JDW229	CAGGTCGACTCTAGAGGATCGCGGCCCAGGCCAAAGGC	
JDW297	ATTCGAGCTCGGTACCCGGGAAGCCTCCAAGACCCTCAG	TS1 ΔPP_5003-6

JDW298	TCCAGCAGGCCTACGACGCTCCGTTGTC	
JDW299	AGCGTCGTAGGCCTGCTGGAGATGTAGTG	TS2 ΔPP_5003-6
JDW300	CAGGTCGACTCTAGAGGATCGCGAACTTGAAGAAGCCTTC	
JDW305	ATTCGAGCTCGGTACCCGGGACATCGAGGATTGCGCTG	TS1 Δ*secG*
JDW306	AACTGAACAACGGGTTTCAAGTAGTAGTATTGC	
JDW307	TTGAAACCCGTTGTTCAGTTTCCTGCGG	TS2 Δ*secG*
JDW308	CAGGTCGACTCTAGAGGATCATTGATGGCCTGGCAGGTAAAG	
YA89	TAACAGGGTAATCTGAATTCGTCCAGGCTCTGCGCCCCG	TS1 Δ*gcdR*, also used for SNV
YA90	GTTGACGTACCCCTGTAGTCAATTATTTTAAACACCTACAGATGTATGTATATGTCGC	
YA91	GACTACAGGGGTACGTCAACCTCACTTGTAAG	TS2 Δ*gcdR*, also used for SNV
YA92	GCCTGCAGGTCGACTCTAGAGAACACATTGTCCATGAC	
YA95	CCCTGTAGTCAATTATTTTAAACACCTACAGATGTATGTATATGTCGC	TS1 Δ*gcdR* and P_{14f} integration
YA96	TAAAATAATTGACTACAGGGTTAATTAAGCCCGTTGACATGACATGGTTTTGAGGGTATAATGTGGCGACCTAGGGTACGTCAACCTCACTTGTAAG	TS2 Δ*gcdR* and P_{14f} integration
YA145	GCAACCATCCCCGAGCAATACG	qPCR amplicon *gcdH*
YA146	ATCACCAGCGACGACTGCACAC	
SEM_MM_99	AGGATCCUCTAGAGTCGACCTGCAGGC	pS648 amplification
SEM_MM_101	ATGTTTTUCCTCCTTCATGACTCCATTATTATTGTTTC	pS648 amplification
SEM_MM _97	AAAAACAUATGGCGACCGGCAAAGGC	sRBS + *phaC* amplification
SEM_MM_103	ATGTTTTTCCUCCTGAATTCTCATGCCTTGGCTTTGACGTATCGC	*phaC* amplification
SEM_MM_104	AGGAAAAACAUATGACTGACGTTGTCATCGTATC	sRBS + *phaA* amplification
SEM_MM_105	ATGTTTTUCCTCCTGAATTCTTATTTGCGCTCGACTGCC	*phaA* amplification
SEM_MM_106	AAAAACAUATGACTCAGCGCATTGCGTAT	sRBS + *phaB* amplification
SEM_MM_98	AGGATCCUCAGCCCATATGCAGGCCG	*phaB* amplification
SEM_MM-121	ATTAAACGUTCAAAAAACAATAGAGGAGACTGAATTTTCAGACACG	ChnR/P_{chnB} amplification
SEM_MM_79	ATCCCCGGGUACCGAGCTCGAATTCGCGCGGC	ChnR/P_{chnB} amplification
SEM_MM_ 80	ACCCGGGGAUCCTAAGGAGGAAAAACATATGGCGACC	(sRBS)*phaCAB* amplification
SEM_MM_120	AGCTTGCAUTCAGCCCATATGCAGGCC	(sRBS)*phaCAB* amplification

Table S3: Plasmids used in this work

Plasmids	Description	Reference
**pRK600**	Cm^R, *oriV*(ColE1), *tra*$^+$ *mob*$^+$ of RK2	Keen et al. (1988)
**pRK2013**	Km^R, *oriV*(RK2/ColE1) -*mob*$^+$ *tra*$^+$	Figurski and Helinski (1979)
**pBBFLP**	Helper plasmid used for antibiotic markers excision in *P. putida* strains; *oriV*(pBBR1) *oriT*(RK2) *mob*$^+$ λP_R::FLP λ(cI857) *sacB tet*, Tc^R	de las Heras et al. (2008)
**pEMG**	Km$_R$, *oriV*(R6K), *lacZ* α with two flanking I-SceI sites	Martínez-García and de Lorenzo, (2011)
**pSNW2**	pEMG with *msfGFP*,	Volke et al., (2020)
**pSW-2**	Gm^R, *oriV*(*RK2*), *xylS*, Pm I-SceI (transcriptional fusion of I-SceI to Pm)	Martínez-García and de Lorenzo (2011)
**pEMG_PP_2051**	pEMG harboring flanking sequences for ΔPP_2051	Li et al., (2020)
**pEMG_*dcaAKIJP***	pEMG harboring flanking sequences for Δ*dcaAKIJP*	This study

Plasmids	Description	Reference
**pEMG_*ttgB***$^{\Delta 4bp}$	pEMG harboring flanking sequences for *ttgB*$^{\Delta 4bp}$	This study
**pEMG_PP_2046**^{A247T}	pEMG harboring flanking sequences for PP_2046^{A247T}	This study
**pEMG_PP_2790**^{A222V}	pEMG harboring flanking sequences for PP_2790^{A222V}	This study
**pEMG_PP_5423**^{R29P}	pEMG harboring flanking sequences for PP_5423^{R29P}	This study
**pEMG_*secG***^{G70R}	pEMG harboring flanking sequences for *secG*^{G70R}	This study
**pEMG_*secG***	pEMG harboring flanking sequences for Δ*secG*	This study
**pEMG_PP_5003-6**	pEMG harboring flanking sequences for ΔPP_5003-6	This study
**pS648::(sRBS)*phaCAB***	introduce synthetic RBSs upstream PHB operon	This study
**pS341·PHA**	For amplification of *phaCAB*	Durante-Rodríguez et al., (2018)
**pS6311·PHB**	Derivative of pSEVA631 harboring a synthetic operon for inducible expression of the *phaCAB* genes from *C. necator*	This study
**pSNW2_*gcdR***	pSNW2 harboring flanking sequences for Δ*gcdR*	This study
**pSNW2_*gcdR*_**P_{14f}	pSNW2 harboring flanking sequences for Δ*gcdR* and Integration of P_{14f}	This study
**pSNW2_*gcdR***^{G148D}	pSNW2 harboring flanking sequences for *gcdR*^{G148D}	This study
**pSNW2_*gcdR***^{G154D}	pSNW2 harboring flanking sequences for *gcdR*^{G154D}	This study
